# Adherence to a Mediterranean Diet and Thyroid Function in Obesity: A Cross-Sectional Apulian Survey

**DOI:** 10.3390/nu12103173

**Published:** 2020-10-16

**Authors:** Roberta Zupo, Fabio Castellana, Francesco Panza, Luisa Lampignano, Isanna Murro, Carmen Di Noia, Vincenzo Triggiani, Gianluigi Giannelli, Rodolfo Sardone, Giovanni De Pergola

**Affiliations:** 1 Population Health Unit—“Salus in Apulia Study”—National Institute of Gastroenterology—Research Hospital, IRCCS “S. De Bellis”, Castellana Grotte, 70013 Bari, Italy; castellanafabio@hotmail.it (F.C.); f_panza@hotmail.com (F.P.); luisalampignano@gmail.com (L.L.); rodolfo.sardone@irccsdebellis.it (R.S.); gdepergola@libero.it (G.D.P.); 2 Neurodegenerative Disease Unit, Department of Basic Medicine, Neuroscience, and Sense Organs, University of Bari Aldo Moro, 70124 Bari, Italy; 3 Department of Biomedical Science and Human Oncology, School of Medicine, Policlinico, University of Bari, 70124 Bari, Italy; murro.isanna3@gmail.com (I.M.); carmen.dinoia@libero.it (C.D.N.); 4 Section of Internal Medicine, Geriatrics, Endocrinology and Rare Disease, Interdisciplinary Department of Medicine, School of Medicine, University of Bari, 70124 Bari, Italy; vincenzo.triggiani@uniba.it; 5 Scientific Direction, National Institute of Gastroenterology “Saverio de Bellis”, Research Hospital, Castellana Grotte, 70013 Bari, Italy; gianluigi.giannelli@irccsdebellis.it

**Keywords:** mediterranean diet, thyroid hormones, obesity, Italy

## Abstract

Much research suggests that Mediterranean eating habits and lifestyle contribute to counteract the risk of chronic diseases while promoting longevity, but little information is available on the effects of the Mediterranean diet (Med-Diet) on thyroid function, particularly among overweight/obese subjects. Nevertheless, consistent data reported a slight increase in serum levels of the thyroid-stimulating hormone (TSH) and a higher rate of conversion of thyroxine (T4) to triiodothyronine (T3) in obesity. This cross-sectional study was aimed at investigating the relationship between adherence to the Med-Diet and circulating thyroid hormones in a cohort of overweight/obese subjects from Apulia (Southern Italy). **Methods:** We studied 324 consecutive outpatient subjects (228 women and 96 men, age range 14–72 years) taking no drug therapy and showing normal levels of thyroid hormones, but complicated by overweight and obesity (body mass index (BMI) ≥ 25 Kg/m^2^). The PREDIMED (PREvención con DIeta MEDiterránea) questionnaire was cross-sectionally administered to assess the adherence to the Med-Diet, and hormonal, metabolic, and routine laboratory parameters were collected. **Results:** Higher adherence to Med-Diet was found to be inversely related to free T3 (*p* < 0.01) and T4 (*p* < 0.01) serum levels. Considering each item in the PREDIMED questionnaire, people consuming at least four spoonfuls of extra-virgin olive oil (EVOO) per day, as well as those consuming at least two servings of vegetables per day, had lower free T3 levels (*p* 0.033 and *p* 0.021, respectively). Furthermore, consuming at least four spoonfuls of EVOO per day was found to be associated to lower free T4 serum concentrations (*p* 0.011). Multinomial logistic regression models, performed on tertiles of thyroid hormones to further investigate the relationship with Med-Diet, corroborated the significance only for free T4. **Conclusion:** Increased adherence to the Med-Diet was independently associated to a slightly reduced thyroid function, but still within the reference range for free T3 and T4 serum levels. This first finding in this field opens up a research line on any underlying biological interplay.

## 1. Introduction

The traditional Mediterranean diet (Med-Diet) is the heritage of millennia of cultures, food patterns, and customs of the inhabitants of the entire Mediterranean basin, characterized by the main consumption of typical agricultural foods (i.e., olive oil, fruits, vegetables, and cereals) produced by local, small and medium-sized rural enterprises. As a demonstration of universally appreciated and approved culinary practices making part of a broader popular culture where the quality, simplicity, and healthiness of native agricultural products are combined with traditional food practices, territoriality, and biodiversity, according to seasonal production, the UNESCO (United Nations Educational, Scientific and Cultural Organization) has recognized this diet as intangible cultural heritage [1]. By and large, the Med-Diet stands out for the high amount of vegetables, fruits, legumes, extra virgin olive oil (EVOO), nuts, and complex carbohydrates, versus a moderate intake of fish and poultry, and a low intake of dairy products, red meat, processed meats, and sweets. Also, a moderate consumption of alcohol (primarily red wine) during meals [2,3,4,5]. On this basis, the Med-Diet has been referred to as a high-fat diet (approximately 40% of total energy intake), given the wide consumption of EVOO as major cooking fat in place of animal sources (i.e., butter), that is also high in complex carbohydrates and fibers, mostly derived from vegetables and fruits. Altogether, this dietary pattern provides a low quantity of saturated fatty acids and a high amount of antioxidants and fibers, as well as a considerable intake of monounsaturated fatty acids and n-3 PUFAs (Polyunsaturated Fatty Acids), mainly derived from EVOO. These hallmarks have been described as mostly responsible for the lowest all-cause mortality rates since the 25 years Seven Countries Study was reported [6]. In this respect, based upon lots of evidence, the Med-Diet has been included among the highly beneficial dietary approaches to avert major noncommunicable diseases, such as cardiovascular diseases (CVDs) [7,8,9], cancer [10], type 2 diabetes mellitus (T2DM), and chronic kidney diseases (CKDs) [11]. Regarding current knowledge on cardiovascular (CV) risk, a greater adherence to Med-Diet has been shown to counter the onset of leading CV risk factors including obesity [7], hypertension [12], dyslipidaemia [8], endothelial dysfunction [13], insulin resistance [7], a prothrombotic state, and low-grade systemic inflammation [14], while fostering the increase of circulating high-density lipoprotein (HDL) cholesterol [8], vitamin D [15], and antiatherogenic lipid subclasses [16]. The PREDIMED (PREvención con DIeta MEDiterránea) multicenter, randomized, primary prevention trial assessing the long-term effects of the Med-Diet on CV clinical events has attributed a better CV health to the abundance of unsaturated fats from natural plant sources [17].

Accumulated evidence postulates that metabolic disturbances and thyroid dysfunction are closely linked in the clinical context. Indeed, the thyroid gland is the main center of metabolic regulation and its homeostasis may substantially affect human health during the lifetime. Basal metabolic rate (BMR), heat production, and oxygen consumption depend on thyroid hormones release and activity. Hence, the most common issue linked to thyroid dysfunction is doubtless obesity. Free triiodothyronine concentrations have been shown to be directly associated with abdominal adiposity, independently of insulin resistance, possibly as a compensatory mechanism for a thermogenic defect [18]. As for diet, besides the well-known involvement of iodine dietary sources, some reports in literature have described goitrogenic foods that can affect thyroid function by inhibiting the synthesis of thyroid hormones, resulting in hypothyroidism and goiter. Regarding iodine, which is especially high in iodized salt, cereals, seafood, beef, poultry, pudding mixes, milk, and dairy products, much research has described a possible involvement in triggering thyroid disturbances [19], in cases of both low and excessive dietary exposure. Soy products and cruciferous vegetables have been described as goiterogenic dietary sources [20,21], since they have a higher amount of a cyclic thiocarbamate chemical compound, named goitrin, that acts as a disruptor of thyroid hormones production by interfering with the iodine metabolism in the thyroid gland. This latter leads the pituitary to release thyroid-stimulating hormones, which in turn promote the growth of thyroid tissue, eventually leading to goiter. By contrast, selenium and zinc have been elsewhere described as beneficial for thyroid function, in terms of preventing cell damage and premature aging [22,23]. Lastly, limited preliminary works have found lower vitamin D and calcium levels to be significantly associated with hypothyroidism, but further studies are needed to corroborate any causal link [24]. Unlike the evidence concerning single nutrients, there is no definite knowledge about any effect of specific dietary patterns or traditional dietary lifestyles on thyroid function. In fact, surprisingly, no studies have yet investigated the possible influence of the Mediterranean diet on thyroid hormones, despite the growing awareness about the many effects played by nutritional habits on the state of health. On this basis, our study aimed at investigating the association between the level of adherence to the Med-Diet model, assessed by the PREDIMED score, and circulating levels of thyroid-stimulating hormone (TSH), free triiodothyronine (T3,) and thyroxine (T4).

## 2. Materials and Methods

### 2.1. Study Population and Design

From January 2018 to December 2019, 324 consecutive patients (228 women and 96 men, aged 14–72 years) were recruited at the “Population Health Unit” of the National Institute of Gastroenterology “S. de Bellis” Research Hospital, Castellana Grotte, Apulia, Italy. All data were collected according to a cross-sectional design. Inclusion criteria were overweight or obesity (body mass index (BMI) ≥ 25 Kg/m^2^), in subjects taking no supplements or medication, including oral contraceptives or medicines for osteoporosis. No upper limit was used for BMI values in this study. Exclusion criteria were any history of endocrinological diseases (diabetes mellitus, hypo or hyperthyroidism, hypopituitarism, etc.), chronic inflammatory diseases, stable hypertension, angina pectoris, stroke, transient ischemic attack, atrial fibrillation, heart infarction, congenital heart disease, any malignancies, renal and liver failure, and inherited thrombocytopenia. The study protocol (ClinicalTrials.gov Identifier: NCT04327375) met the principles in the Declaration of Helsinki and was approved by the Ethics Committee of National Cancer Research Centre “Giovanni Paolo II”, Bari, Italy (protocol n. 234 CE 21/06/2019). All participants gave informed consent prior to enrolment in accordance with the Helsinki Declaration of 1964 and subsequent revisions.

### 2.2. Assessment of Adherence to Med-Diet

Two senior nutritionists (RZ and FC) administered a validated 14-item Mediterranean diet adherence screener, previously validated by the multicenter clinical trial PREDIMED (PREvención con Diet Mediterránea) [25] (Supplementary Figure S1), that we used in our previous study [15]. The questionnaire includes 14 closed questions (yes/no) investigating the frequency of intake of foods characterizing the Mediterranean model. Each of the 14 items is scored 1 or 0, depending on whether participants adhere to each Med-Diet component or not. The final score (from 0 to 14) of each subject indicates the level of adherence to the Med-Diet; a score ≥7 is considered a good level of adherence to the Med-Diet [25] and we used it as cut-off to determine the adherence to Med-Diet in this study.

### 2.3. Clinical Examination and Laboratory Biomarkers Collection

Hormonal, metabolic, and routine biochemistry parameters were closely examined in all subjects at the time of enrollment. A brief interview was administered by a senior physician, including questions on medical history and lifestyle. Using the OMRON M6 Automated Blood Pressure Monitor, extemporaneous diastolic (DBP) and systolic blood pressure (SBP) were calculated in outpatients in a sitting position after at least 10 min of rest, at least three separate times.

Blood samples were drawn between 08:00 and 09:00h a.m., after overnight fasting. Fasting plasma glucose (FPG), insulin, total cholesterol, high- and low-density lipoprotein (HDL, LDL) cholesterol, and triglycerides serum levels were assayed. Serum insulin concentrations were measured by radioimmunoassay (Behring, Scoppito, Italy), and all samples were analyzed in duplicate. Plasma glucose was determined using the glucose oxidase method (Sclavus, Siena, Italy), while the concentrations of plasma lipids (triglycerides, total cholesterol, HDL cholesterol) were quantified by automated colorimetric method (Hitachi; Boehringer Mannheim, Mannheim, Germany). LDL cholesterol was calculated using the Friedewald equation [26]. TSH, free triiodothyronine (FT3), and free thyroxine (FT4) serum concentrations were measured by a competitive luminometric assay based on the SPALT (solid-phase antigen luminescence technique) principle (LIAISON FT3, FT4, TSH, DiaSorin, Saluggia, Italy). Serum uric acid was measured by the URICASE/POD method implemented in an autoanalyzer (Boehringer Mannheim, Mannheim, Germany). Serum intact PTH (Parathyroid Hormone) levels were measured using a standard enzyme-linked immunosorbent PTH immunoradiometric assay (IBL International GmbH, Hamburg, Germany) with 1.0 pg/mL as the lower limit of detection (Roche Diagnostics Instrumental material). Creatinine was measured by an automated system (UniCel Integrated Workstations DxC 660i, Beckman Coulter, Fullerton, CA, USA). Insulin resistance was assessed using the homeostasis model assessment—insulin resistance (HOMA-IR) [27].

### 2.4. Anthropometric Assessment

Two qualified nutritionists (RZ and FC), equipped for equal calculation results, performed clinical procedures. All anthropometric measurements were taken with participants dressing without shoes and in lightweight clothes. Variables were all collected at the same time between 7:00 and 10:00 a.m. in the morning, after overnight fasting. Height was measured to the nearest 0.5 cm using a wall-mounted stadiometer (Seca 711; Seca, Hamburg, Germany). Body weight was determined to the nearest 0.1 kg using a calibrated balance beam scale (Seca 711; Seca, Hamburg, Germany). BMI was calculated by dividing body weight (Kg) by the square of height (m^2^) and classified according to World Health Organization criteria for normal weight (18.5–24.9 kg/m^2^), overweight (25.0–29.9 kg/m^2^), grade I obesity (30.0–34.9 kg/m^2^), grade II obesity (35.0–39.9 kg/m^2^), and grade III obesity (≥40.0 kg/m^2^) [28]. Waist circumference (WC) was measured at the narrowest part of the abdomen, or in the area between the tenth rib and the iliac crest (minimum circumference).

### 2.5. Statistics

We performed statistical analysis of baseline variables, expressed as mean ± standard deviation (SD), median, and range for continuous variables, and proportion (%) for the frequency of categorical variables. The normality of distribution was assessed for each variable using Shapiro’s test. Spearman’s correlation matrix was built for all continuous biochemical and anthropometric variables to check for interrelated variables, to avoid collinearity effects in the regressive models.

The entire sample was divided into two groups using a 7-point threshold of the PREDIMED score to categorize the adherence to Med-Diet (low adherence if ˂7, high adherence if ≥7). A Wilcoxon sum rank test was performed to assess any difference between collected variables in the two groups. Age (continuous variable), gender (dichotomous variable), systolic and diastolic BP (continuous variables), and BMI (continuous variable) were used as major covariates when performing regression models. Three multinomial logistic regression models were used to ascertain the association between free thyroid hormones, subdivided into tertiles, and PREDIMED score (dichotomous variable). The adjustments for each logistic regression model are reported as endnotes at each table. Lastly, we stratified thyroid hormones across tertiles in order to corroborate the different distribution of values between less/more adherent subjects. A *p*-value less than or equal to 0.05 was considered statistically significant, with 95% confidence intervals.

## 3. Results

The whole sample (N = 324) featured a majority of women (70.4%). Mean age was 41.68 ± 13.25 years. Mean BMI was 33.17 ± 5.4 Kg/m^2^. Table 1 summarizes the general, anthropometric, hormonal, metabolic, and routine laboratory parameters of the enrolled subjects, expressed as mean ± SD, median, and range for continuous variables, and as percentage (%) for proportions. A Spearman’s correlation matrix (not shown) between the PREDIMED score and all continuous biochemical and anthropometric variables showed that a higher adherence to Med-Diet was significantly related to lower circulating FT3 (*p* 0.019), FT4 (*p* 0.001), and uric acid (*p* 0.047) serum levels. Moreover, Spearman’s correlation matrix did not show high co-linearity between collected variables, highlighting that older subjects showed a greater adherence to Med-Diet (*p* 0.014). No differences in other collected variables were found across the two categories of adherence to the Med-Diet.

After analyzing the relationship between free thyroid hormones (FT3 and FT4) and each of the PREDIMED items using the Wilcoxon sum rank test (not shown), we found that the use of at least four tablespoonfuls of EVOO per day and the consumption of a serving of vegetables at least twice a day were the only features of the Med-Diet that showed a significant negative correlation with FT3 (*p* 0.033 and *p* 0.021, respectively); moreover, the consumption of a serving of vegetables at least twice a day was the only Med-Diet feature showing a significant inverse association with FT4 serum levels (*p* 0.011).

After performing multinomial logistic regression models, the adherence to the Med-Diet, as evaluated by the PREDIMED score, was a significant predictive factor of a decreased thyroid activity, as evaluated by tertiles of free T3 and T4. Raw models maintained the significant association, while in the semi- and full-adjusted kept significance only for free T4 (Table 2). The different distribution of thyroid hormones between less/more adherent groups was corroborated also after stratifying the sample in tertiles (Table 3). Subjects in the higher tertile for FT3 and FT4 serum levels were significantly less adherent to the Med-Diet (*p* 0.03 and *p* 0.02, respectively). In addition, subjects in the first tertile for FT4 serum levels had a significantly higher adherence to the Med-Diet (*p* < 0.01).

## 4. Discussion

In our outpatient-based sample of three hundred and twenty-four subjects with thyroid function within the reference range, we observed a cross-sectional inverse association between PREDIMED score and T3 and T4 circulating hormones. No significant association was found with serum TSH concentrations.

These results, even if soiled by a cross-sectional clinically based setting, would suggest a possible inhibitory effect exerted by the Med-Diet on thyroid function. Moreover, while adherence to Med-Diet was significantly and negatively correlated with free thyroid hormones, it was not associated with TSH levels, thus excluding an adaptation of the hypothalamus–pituitary axis or a real decrease of the thyroid gland function. On the other hand, we cannot exclude that a higher adherence to Med-Diet increases the tissue and organ sensitivity to thyroid hormones, with the slightly lower concentrations of free thyroid hormones representing an adaptation to a higher adherence to Med-Diet. Besides, since the inverse association between Med-Diet and thyroid hormones was corroborated regardless of gender, age, and BMI, we should exclude a direct effect on this relationship. As for age, the adherence to Med-Diet was significantly and positively related to age, younger subjects being less inclined to adhere to a Mediterranean dietary setting; this finding brings to light a gradual trend of the new generations to move away from traditional dietary patterns, thus emphasizing the phenomenon of “modern” eating behaviors [29].

A tough question is whether the relationship between Med-Diet and thyroid function is attributable to the traditional features of Mediterranean lifestyle, or whether it results from typical regional shades of the original Med-Diet model [30]. In this regard, after analyzing the relationship between free thyroid hormones and each of the PREDIMED items, we found that the use of at least four tablespoonfuls of EVOO per day and the consumption of a serving of vegetables at least twice a day were the only items showing a significant negative association with FT3. Moreover, the consumption of a serving of vegetables at least twice a day was the only factor showing a significant inverse association with serum FT4 levels. However, after performing multiple regression analyses including EVOO and the consumption of vegetables as regressors, we found that only FT4 maintained the inverse association to Med-Diet. This suggests that Med-Diet, as a holistic lifestyle approach, could likely be responsible for lowering circulating thyroid hormone levels. Several key questions remain to be solved with further research, so as to make clinically relevant judgments.

Nevertheless, given the well-proven positive implications of the Med-Diet on the gut microbiota composition, which is related to several metabolic disorders, a possible involvement of its fermentative metabolites should not be excluded [31]. Indeed, the Med-Diet is abundant in fruit, vegetables, and legumes, which are foods laden with complex carbohydrates. The latter are fermented by healthy gut microbiota, in turn producing a multitude of short-chain fatty acids (such as butyrate, propionate, and acetate) with all their purported benefits for human health, including thyroid function [32].

Some limitations of this study should be considered. Because of the cross-sectional setting, the direction of any causal relationship could not be established, so our data provide a description rather than an explanation. Prospective studies are needed to clarify a causal relationship. Moreover, the study has a selection bias due to the absence of healthy volunteers, as all subjects were recruited at a hospital clinic, and thus they most likely had some reason to present there. This means that our results cannot be generalized but only applied to a population with the same clinical characteristics. Also, data on iodine intake and/or excretion are lacking, so we cannot justify our findings based on other dietary features. We can only consider previous data reporting the presence of mild-to-moderate iodine deficiencies in Southern Italy [33].

A strong point is that we examined only individuals taking no medication, thus avoiding a possible interference with biomarkers assays and hence investigated outcomes. However, this selection method may have excluded a percentage of younger or older subjects, given the wide utilization of oral contraceptives and supplementation in those age groups, respectively. Furthermore, the investigation was carried out in a typical Mediterranean region, Apulia, in the South of Italy. To the best of our knowledge, no observation in such a setting has ever been recorded before, and our results, although affected by some limitations, therefore stand out for originality in this field.

## 5. Conclusions

This research, conducted on a reasonably large sample of subjects belonging to a typical Mediterranean region and sharing a condition of overweight or obesity, shows a strong negative association between Med-Diet adherence and free T3 and T4 serum levels. This may be a very useful starting point to get researchers interested in this unexplored area. Currently, we can only assume that Med-Diet, as an overall healthy lifestyle model, may slightly inhibit the production of thyroid hormones, but without significantly changing thyroid function.

## Figures and Tables

**Table 1:** Description of the whole sample divided by adherence to Mediterranean diet (Med-Diet) (*N* = 324).

	*PREDIMED Score (<7)*		*PREDIMED Score (≥7)*		
	*Mean ± SD*	*Median (Min to Max)*	*Mean ± SD*	*Median (Min to Max)*	p Value *
Proportions	59 (18.20)	--	265 (81.80)	--	
Age (years)	38.034 ± 13.165	37 (19 to 72)	42.494 ± 13.157	45 (14 to 71)	**0.014**
*Gender*					
*Male*	23 (24.00)	--	73 (76.00)	--	0.08 ^χ2^
*Female*	36 (15.80)	--	192 (72.50)	--	
BMI (Kg/m^2^)	34.334 ± 5.579	33.5 (26.1 to 47.3)	32.914 ± 5.374	31.5 (24 to 49.3)	0.073
Waist Circumference (cm)	111.661 ± 15.952	114 (81 to 150)	107.536 ± 12.582	106 (79 to 147)	0.067
SBP (mmHg)	129.186 ± 18.1	130 (86 to 180)	128.649 ± 16.122	130 (90 to 172)	0.686
DBP (mmHg)	85.237 ± 12.934	85 (55 to 116)	83.532 ± 11.409	84 (56 to 120)	0.277
FBG (mg/dL)	88.776 ± 10.122	87.5 (70 to 118)	89.782 ± 12.884	88 (65 to 143)	0.744
Insulin (μUI/mL)	14.991 ± 9.367	12 (4.3 to 46)	14.201 ± 9.55	11.5 (2.4 to 67)	0.471
HOMA index	3.348 ± 2.262	2.77 (0.74 to 11.1)	3.189 ± 2.266	2.51 (0.52 to 14.5)	0.479
HbA1c (%)	5.359 ± 0.423	5.375 (4.6 to 6.9)	5.374 ± 0.485	5.35 (4 to 8.9)	0.982
Total cholesterol (mg/dL)	190.702 ± 39.88	190 (125 to 298)	194.695 ± 38.747	192 (51 to 310)	0.467
HDL cholesterol (mg/dL)	48.526 ± 14.345	44 (24 to 89)	52.092 ± 13.395	50 (27 to 116)	0.022
LDL cholesterol (mg/dL)	123.982 ± 35.071	122 (69 to 262)	122.494 ± 33.396	118 (32 to 221)	0.899
Triglycerides (mg/dL)	109.797 ± 60.549	101 (39 to 400)	105.781 ± 62.04	93 (31 to 541)	0.451
TSH (microUI/mL)	1.786 ± 0.993	1.6 (0.38 to 4.31)	1.927 ± 1.055	1.67 (0.29 to 6.37)	0.321
FT3 (pg/mL)	3.122 ± 0.397	3.105 (2.1 to 3.9)	2.993 ± 0.415	2.995 (1.64 to 4.6)	**0.019**
FT4 (pg/mL)	10.911 ± 1.508	10.75 (8 to 16.5)	10.23 ± 1.341	10.1 (6.8 to 14.5)	**0.001**
PTH (pg/mL)	32.367 ± 17.081	28.25 (5.2 to 98.6)	31.32 ± 12.893	29 (7.46 to 76.7)	0.928
Uric acid (mg/dL)	5.098 ± 1.651	4.9 (2.1 to 10.4)	4.603 ± 1.393	4.5 (2.1 to 9.4)	**0.047**
Creatinine (mL/min)	0.766 ± 0.15	0.755 (0.48 to 1.16)	0.768 ± 0.161	0.75 (0.46 to 1.26)	0.852

* Wilcoxon sum rank test, ^ꭓ2^ Chi squared test. Data are shown as mean ± SD (Standard Deviation), median and range for continuous variables and as (%) for proportions. Significance shown in bold. BMI: body mass index; SBP: systolic blood pressure; DBP: diastolic blood pressure; FBG: Fasting Blood Glucose; HOMA: Homeostatic Model Assessment for Insulin Resistance; HbA1c: Hemoglobin A1c; HDL cholesterol: high-density lipoprotein cholesterol; LDL cholesterol: low-density lipoprotein cholesterol; TSH: thyroid-stimulating hormone; FT3: free triiodothyronine; FT4: free thyroxine; PTH: Parathyroid Hormone.

**Table 2:** Multinomial logistic regression models on FT4 and FT3 tertiles as dependent variables.

		FT4 Tertiles			FT3 Tertiles		
		Raw Models					
		OR	CI 95%	*p* Value	OR	CI 95%	*p* Value
PREDIMED score (>7)	2nd tertile	**0.35**	**0.15 to 0.79**	**0.01**	0.75	0.35 to 1.60	0.46
	3rd tertile	**0.26**	**0.11 to 0.61**	**<0.01**	0.46	0.22 to 0.94	**0.03**
		Semi-adjusted models *					
	2nd tertile	**0.34**	**0.14 to 0.78**	**0.01**	0.87	0.40 to 1.89	0.72
	3rd tertile	**0.26**	**0.11 to 0.59**	**<0.01**	0.53	0.25 to 1.12	0.09
		Fully-adjusted models **					
	2nd tertile	**0.33**	**0.12 to 0.93**	**0.03**	1.10	0.41 to 2.89	0.84
	3rd tertile	**0.30**	**0.11 to 0.83**	**0.02**	0.61	0.24 to 1.57	0.31

Significance shown in bold. * corrected for age, gender, SBP (Systolic Blood Pressure), DBP (Diastolic Blood Pressure), and BMI (Body Mass Index). ** corrected for semi-adjusted plus olive oil consumption (>4 servings/day) and vegetables consumption (>2 servings/day). Abbreviations: FT3 (free triiodothyronine); FT4 (free thyroxine). OR: Odds Ratio; CI: Confidence Interval; PREDIMED: (PREvención con DIeta MEDiterránea).

**Table 3:** Distribution across thyroid hormones tertiles according to the PREDIMED (PREvención con DIeta MEDiterránea) score.

		PREDIMED Score <7	PREDIMED Score ≥7	*p* Value *
TSH (microUI/mL)	Q1 (0.29–1.28)	20 (34.50)	86 (33.30)	0.86
	Q2 (1.29–2.16)	20 (34.50)	85 (32.90)	0.82
	Q3 (2.17–6.37)	18 (31.00)	87 (33.70)	0.69
FT3 (pg/mL)	Q1 (1.64–2.83)	14 (24.10)	90 (35.40)	0.09
	Q2 (2.84–3.17)	18 (31.00)	87 (34.30)	0.63
	Q3 (3.18–4.60)	26 (44.80)	77 (30.30)	**0.03**
FT4 (pg/mL)	Q1 (6.80–9.72)	9 (15.50)	95 (37.40)	**<0.01**
	Q2 (9.73–10.80)	23 (39.70)	85 (33.50)	0.37
	Q3 (10.81–16.50)	26 (44.80)	74 (29.10)	**0.02**

Data shown as count and (%). * Chi-squared test. Abbreviations: TSH (thyroid-stimulating hormone); FT3 (free triiodothyronine); FT4 (free thyroxine).

## Supplementary Materials

The following are available online at https://www.mdpi.com/2072-6643/12/10/3173/s1. Figure S1: Quantitative score (14-item) of adherence to the Mediterranean Diet.
