# Supplementary material for: Adherence to a Mediterranean Diet and Thyroid Function in Obesity: A Cross-Sectional Apulian Survey
Source: Nutrients. 2020 Oct 16;12(10):3173. doi: 10.3390/nu12103173 (PMC7603040; doi:10.3390/nu12103173)
Supplement: Supplementary file 1 [file nutrients-12-03173-s001.pdf]

Figure S1. Quantitative score (14-item) of adherence to the Mediterranean Diet.

	Frequency[1]
1. Do you use olive oil as the principal source of fat for cooking?	Yes
2. How much olive oil do you consume per day (including that used in frying, salads, meals eaten away from home, etc.)?	≥4 Tbsp[5]
3. How many servings of vegetables do you consume per day? Count garnish and side servings as 1/2 point; a full serving is 200 g.	≥2
4. How many pieces of fruit (including fresh-squeezed juice) do you consume per day?	≥3
5. How many servings of red meat, hamburger, or sausages do you consume per day? A full serving is 100–150 g.	<1
6. How many servings (12 g) of butter, margarine, or cream do you consume per day?	<1
7. How many carbonated and/or sugar-sweetened beverages do you consume per day?	<1
8. Do you drink wine? How much do you consume per week?	≥7 cups[6]
9. How many servings (150 g) of pulses do you consume per week?	≥3
10. How many servings of fish/seafood do you consume per week? (100–150 g of fish, 4–5 pieces or 200 g of seafood)	≥3
11. How many times do you consume commercial (not homemade) pastry such as cookies or cake per week?	<2
12. How many times do you consume nuts per week? (1 serving = 30 g)	≥3
13. Do you prefer to eat chicken, turkey or rabbit instead of beef, pork, hamburgers, or sausages?	Yes
14. How many times per week do you consume boiled vegetables, pasta, rice, or other dishes with a sauce of tomato, garlic, onion, or leeks sautÕed in olive oil?	≥2
